# Long‐Distance Avian Migrants Fail to Bring 2.3.4.4b HPAI H5N1 Into Australia for a Second Year in a Row

**DOI:** 10.1111/irv.13281

**Published:** 2024-03-31

**Authors:** Michelle Wille, Robyn Atkinson, Ian G. Barr, Charlotte Burgoyne, Alexander L. Bond, David Boyle, Maureen Christie, Meagan Dewar, Tegan Douglas, Teagan Fitzwater, Chris Hassell, Roz Jessop, Hiske Klaassen, Jennifer L. Lavers, Katherine K.‐S. Leung, Jeremy Ringma, Duncan R. Sutherland, Marcel Klaassen

**Affiliations:** ^1^ Centre for Pathogen Genomics, Department of Microbiology and Immunology, Peter Doherty Institute for Infection and Immunity The University of Melbourne Melbourne Victoria Australia; ^2^ WHO Collaborating Centre for Reference and Research on Influenza Peter Doherty Institute for Infection and Immunity Melbourne Victoria Australia; ^3^ Victorian Wader Study Group Melbourne Victoria Australia; ^4^ Department of Microbiology and Immunology, Peter Doherty Institute for Infection and Immunity The University of Melbourne Melbourne Victoria Australia; ^5^ Northern Australia Quarantine Strategy Department of Agriculture, Fisheries and Forestry Canberra Australian Capital Territory Australia; ^6^ Bird Group The Natural History Museum Tring UK; ^7^ Victorian Ornithological Research Group Inc. Leopold Victoria Australia; ^8^ Australasian Wader Studies Group Melbourne Victoria Australia; ^9^ Friends of Shorebirds SE Carpenter Rocks South Australia Australia; ^10^ Future Regions Research Centre Federation University Australia Berwick Victoria Australia; ^11^ BirdLife Australia Melbourne Victoria Australia; ^12^ Global Flyway Network Broome Western Australia Australia; ^13^ School of Life and Environmental Sciences Deakin University Geelong Victoria Australia; ^14^ Esperance Tjaltjraak Native Title Aboriginal Corporation Esperance Western Australia Australia; ^15^ Gulbali Institute Charles Sturt University Wagga Wagga New South Wales Australia; ^16^ Phillip Island Nature Parks Cowes Victoria Australia; ^17^ School of BioSciences The University of Melbourne Melbourne Victoria Australia

To the Editor,

The current high‐pathogenicity avian influenza (HPAI) H5N1 lineage 2.3.4.4b panzootic is having a profound impact on the poultry industry and wildlife [1, 2]. HPAI H5N1 emerged in poultry in 1996 and has caused outbreaks in wild bird populations episodically since 2005 [3], with the epidemiology of the virus changing substantially with the emergence of new lineages. A novel lineage emerged in 2014 (2.3.4.4), which has diversified and caused substantial mortality, including mass mortality events, of wild birds in 2014, 2016 and 2020–present, marine mammals since 2023, as well as ongoing outbreaks in poultry, globally [3]. Understanding the changing phenotype and viral incursion risk following the emergence of novel lineages of HPAI is of crucial importance for the development of short‐term and long‐term mitigation strategies to protect wildlife, livestock and humans alike.

Wild birds, particularly waterfowl, were initially implicated in the long‐distance spread of HPAI and have been predominantly implicated in the re‐occurring incursions into Europe and Africa [4]. However, recent viral incursions, such as that to the sub‐Antarctic islands, and expansion, such as the movement of HPAI down the spine of South America [5], were driven by seabirds, suggesting that the long‐distance dispersal of lineage 2.3.4.4b HPAI is no longer waterfowl dependent (e.g., [2, 6]).

Lineage 2.3.4.4b has now been detected on all continents except Oceania [7]. HPAI incursion risk to Australia has previously been considered low due to the absence of waterfowl species that migrate beyond the Australio‐Papuan region [8] and from influenza genomic surveillance [9]. In turn, annually, millions of migratory seabirds and shorebirds migrate from Asia and North America to Australia (Figure 1). Some of these species have been shown to be part of the avian influenza reservoir community [10], have been infected by HPAI and potentially survive and move HPAI viruses [11].

**FIGURE 1:**
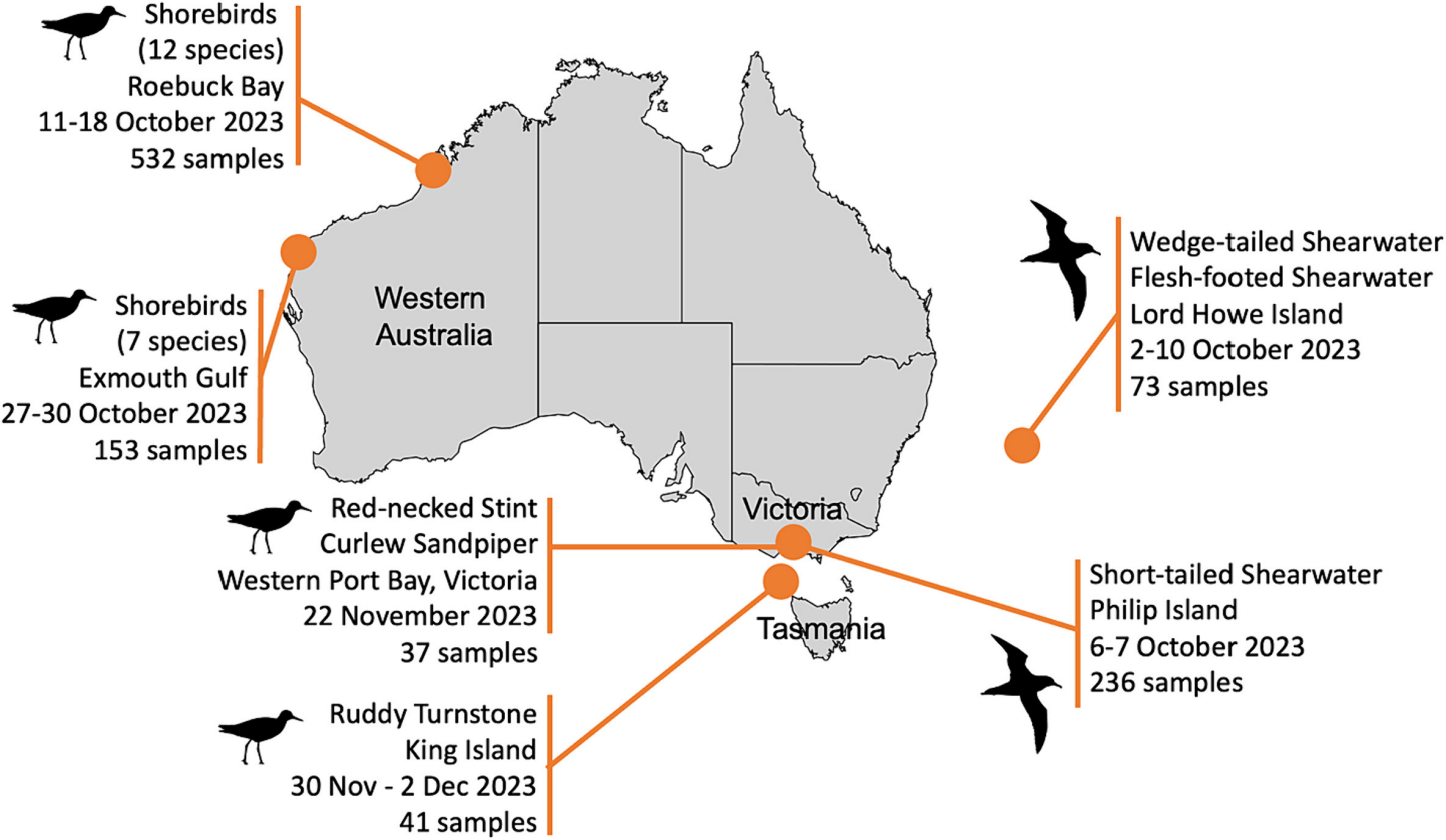
Sampling of incoming migratory birds in Australia for HPAI. All samples were negative for influenza A virus. Birds silhouettes from phylopic.com, distributed under a creative commons attribution.

To reveal whether a viral incursion may have occurred in Australia in 2023 with the arrival of wild migratory seabirds and shorebirds, we investigated 1072 migratory birds of the order *Charadriiformes* and *Procellariiformes*, in October–December 2023. Specifically, we captured and sampled short‐tailed shearwaters (*n* = 236) at a breeding colony on Phillip Island, Victoria, and wedge‐tailed (*n* = 30) and flesh‐footed shearwaters (*n* = 43) at a breeding colony on Lord Howe Island, New South Wales, upon their arrival from the northern Pacific. We also sampled 12 Asian‐breeding migratory shorebird species at major nonbreeding sites in Roebuck Bay (*n* = 532) and Exmouth Gulf (*n* = 153) in Western Australia, Western Port Bay in Victoria (*n* = 37) and on King Island in Tasmania (*n* = 41) (Table 1 and Figure 1).

**TABLE 1:** Charadriiform and procellariiform species targeted for active HPAI surveillance following arrival to Australia after migration from Asia and North America.

Sampling dates	Location	Species	Active infection (qPCR)	Antibody detection (NP ELISA)
6–7 October 2023	Philip Island, Victoria	Short‐tailed shearwater *Ardenna tenuirostris*	0/236	11/226
2–10 October 2023	Lord Howe Island, New South Wales	Flesh‐footed shearwater *Ardenna carneipes*	0/43	0/29
		Wedge‐tailed shearwater *Ardenna pacifica*	0/30	
11–18 October 2023	Broome, Western Australia	Bar‐tailed godwit *Limosa lapponica*	0/43	0/43
		Broad‐billed sandpiper *Limicola falcinellus*	0/13	0/13
		Curlew sandpiper *Calidris ferruginea*	0/95	0/92
		Great knot *Calidris tenuirostris*	0/185	3/185
		Greater sandplover *Charadrius leschenaultii*	0/26	0/26
		Grey‐tailed tattler *Tringa brevipes*	0/3	0/3
		Lesser sandplover *Charadrius mongolus*	0/2	0/2
		Red knot *Calidris canutus*	0/21	2/21
		Red‐necked stint *Calidris ruficollis*	0/126	10/119
		Ruddy turnstone *Arenaria interpres*	0/1	0/1
		Sharp‐tailed sandpiper *Calidris acuminata*	0/10	1/10
		Terek sandpiper *Xenus cinereus*	0/7	0/7
27–30 October 2023	Exmouth Gulf, Western Australia	Bar‐tailed godwit *Limosa lapponica*	0/61	0/61
		Curlew sandpiper *Calidris ferruginea*	0/1	0/1
		Great knot *Calidris tenuirostris*	0/34	0/34
		Greater sandplover *Charadrius leschenaultii*	0/53	0/53
		Grey‐tailed tattler *Tringa brevipes*	0/1	0/1
		Lesser sandplover *Charadrius mongolus*	0/1	0/1
		Red‐necked stint *Calidris ruficollis*	0/2	0/2
22 November 2023	Western Port Bay, Victoria	Curlew sandpiper *Calidris ferruginea*	0/3	0/3
		Red‐necked stint *Calidris ruficollis*	0/34	1/34
30 November–2 December 2023	King Island, Tasmania	Ruddy turnstone *Arenaria interpres*	0/41	7/38

*Note:* The number of active infections with influenza A virus and number of individuals with nuclear‐protein antibodies against influenza A virus are depicted, compared with total number of individuals sampled.

All samples were negative for influenza A virus by qPCR, following [10]. Thirty‐six serum samples tested positive for anti‐NP antibodies using a commercial ELISA (given an S/N cut off of 0.6) [12] (Table 1), which fell within the previously reported seroprevalence of the species that tested positive: great knot, red knot, red‐necked stint, ruddy turnstone, sharp‐tailed sandpiper and short‐tailed shearwater [10]. Thirty‐two sera samples positive by anti‐NP ELISA which had sufficient sample volume remaining were subjected to a haemagglutination inhibition (HI) assay using lineage 2.3.4.4b virus A/Astrakhan/3212/2020(H5N8) and A/American wigeon/South Carolina/22‐000345‐001/2021(H5N1), as well as a Australian lineage LPAI H5N3 Australian lineage virus A/duck/Victoria/0305‐2/2012(H5N3) following [11]. Both 2.3.4.4b antigens were 6:2 recombinant virus on an A/Puerto Rico/8/1934(H1N1)(PR8) backbone with the multibasic cleavage site removed. None of the 32 serum samples tested had evidence for anti‐HA antibodies against either 2.3.4.4b antigen, and one sample (17696) from a short‐tailed shearwater had anti‐HA antibodies against the LPAI H5 Australian lineage virus.

In cases where wild bird mortality events occurred in Australia during this period, all tested birds were negative for HPAI (pers. comm. Wildlife Health Australia).

For Australia, as for other regions in the world, HPAI incursion risk hinges on a combination of factors, including wild bird migration, virus pathogenicity in migratory birds (notably whether birds can migrate while infected) and virus circulation in neighbouring regions. That, like in 2022 [13], there was no incursion of HPAI in Australia in 2023, despite the arrival of millions of migratory birds, could possibly be attributed to two factors. First, while the virus's host reservoir has substantially widened and intensified beyond waterfowl [2, 6, 14], waterfowl are markedly absent from the list of migrants to Australia. HPAI‐infected waterfowl appear to migrate without negative effects, but it is unclear whether migratory culling may prevent the arrival of HPAI‐infected shorebirds and seabirds to Australia. Second, migrants to Australia predominantly use the East Asian Australasian Flyway (EAAF). Aside from few countries, such as Japan, there are few HPAI outbreaks reported in wild birds along the EAAF [2], suggesting a potentially low‐virus exposure potential for long‐distance migratory birds using the EAAF. Further, in Asia, ancestral HPAI H5 lineages, such as 2.3.2.1c in Cambodia or 2.3.2.1a in Bangladesh [3], are endemic and continue to circulate. We speculate that exposure to these more ancestral lineages may potentially result in cross‐protection against 2.3.4.4.b in both poultry and wild birds, serving as a potential explanation why outbreaks of lineage 2.3.4.4b HPAI are remarkably limited in East Asia [2]. None of the migrants tested in this study and in our 2022 study [13] had antibodies against lineage 2.3.4.4b HPAI, and no incursion of lineage 2.3.4.4b HPAI to Australian continent has occurred. These two hypotheses warrant further investigation.

Australia will again enter a high‐risk period when the major bird migrations into the country take place between August and November 2024. Continued surveillance is critical for early detection and rapid response, and as such, we call for enhanced surveillance of Australian wild birds to match heightened incursion risk in the second half of 2024.

## Author Contributions

**Michelle Wille:** Conceptualization; Methodology; Investigation; Formal analysis; Funding acquisition; Visualization; Project administration; Resources; Writing – original draft; Writing – review and editing. **Robyn Atkinson:** Investigation; Writing – review and editing. **Ian G. Barr:** Investigation; Writing – review and editing. **Charlotte Burgoyne:** Investigation; Writing – review and editing. **Alexander L. Bond:** Investigation; Writing – review and editing. **David Boyle:** Investigation; Writing – review and editing. **Maureen Christie:** Investigation; Writing – review and editing. **Meagan Dewar:** Investigation; Writing – review and editing. **Tegan Douglas:** Investigation; Writing – review and editing. **Teagan Fitzwater:** Investigation; Writing – review and editing. **Chris Hassell:** Investigation; Writing – review and editing. **Roz Jessop:** Investigation; Writing – review and editing. **Hiske Klaassen:** Investigation; Writing – review and editing. **Jennifer L. Lavers:** Investigation; Writing – review and editing. **Katherine K.‐S. Leung:** Investigation; Writing – review and editing. **Jeremy Ringma:** Investigation; Writing – review and editing. **Duncan R. Sutherland:** Investigation; Writing – review and editing. **Marcel Klaassen:** Conceptualization; Methodology; Investigation; Formal analysis; Funding acquisition; Visualization; Project administration; Resources; Writing – original draft; Writing – review and editing.

## Ethics Statement

Capture, banding and sampling were conducted under ABBBS authorities 2824 to Tegan Douglas, 2915 to Marcel Klaassen, 8000 to Australasian Wader Studies Group and 8001 to the Victorian Wader Study Group, approval of animal ethics committees of Deakin University (B39‐2019), Natural Resources and Environment Tasmania (AEC Project No. 6/2022‐23 NRE), Philip Island Nature Parks (SPFL20082), Department of Primary Industries and Regional Development Western Australia (WAEC 23‐08‐52) and Charles Stuary University (A22382).

## Conflicts of Interest

The authors declare no conflicts of interest.

### Peer Review

The peer review history for this article is available at https://www.webofscience.com/api/gateway/wos/peer‐review/10.1111/irv.13281.

## Data Availability

All data are available in the manuscript.
